# Muscle attachment site patterns for species determination in West Palaearctic *Wohlfahrtia* (Diptera: Sarcophagidae) of medical and veterinary importance

**DOI:** 10.1038/s41598-019-55127-5

**Published:** 2019-12-16

**Authors:** Senta Niederegger, Kamran Akbarzadeh, Krzysztof Szpila

**Affiliations:** 1 Department of Forensic Entomology, Institute of Legal Medicine at the University Hospital Jena, 07740 Jena, Germany; 2 Department of Medical Entomology & Vector Control, School of Public Health, Tehran University of Medical Sciences, Tehran, Iran; 3 Department of Ecology and Biogeography, Faculty of Biological and Veterinary Sciences, Nicolaus Copernicus University, Lwowska 1, 87-100 Toruń, Poland

**Keywords:** Entomology, Infection

## Abstract

The flesh fly genus *Wohlfahrtia* Brauer & Bergenstamm contains at least six species of medical and veterinary importance. Traditional methods of species identification in specimens of *Wohlfahrtia*, however, are restricted mostly to adult forms. Muscle attachment site (MAS) patterns allow for species determination in larval forms. MAS patterns in third instar larvae of six common West Palearctic species of *Wohlfahrtia* have been analyzed for this study. As in previously investigated Calliphoridae and Sarcophagidae, MAS patterns were found to be species specific. A genus pattern was established to be used as base for comparison in further species determination. For the first time a tool is provided for species identification of such broad range in larvae of *Wohlfahrtia* species. *Wohlfahrtia* patterns are composed of a significantly higher number of MAS than patterns found in *Sarcophaga*. Specifics of the six species analyzed are explained in detail. The larvae of the well-known species *W. magnifica*, an obligate traumatic myiasis agent, had to be excluded from the analysis as a great number of spines on the outside obscure muscle attachment sites on the inside of the cuticle.

## Introduction

The flesh fly genus *Wohlfahrtia* Brauer & Bergenstamm, 1889 contains 24 species distributed mostly in the Palaearctic region^1^. Several of them are of medical and veterinary importance, as they are either facultative or obligate myiasis producers in man or other mammals^2^. The best known is *Wohlfahrtia magnifica*, traumatic myiasis agent, common and widely distributed in the Mediterranean Basin and the Middle East^3^. Necrophagous species of *Wohlfahrtia* may act as facultative parasites but their larvae usually develop in carrion^4–8^. Genus *Wohlfahrtia* belongs to the subfamily Paramacronychiinae traditionally treated as sister to Sarcophaginae^9,10^ but recent molecular studies point to their closer affinity to the subfamily Miltogramminae^11–13^.

Traditional methods of species identification in specimens of *Wohlfahrtia* have been applied mostly to adult forms^14,15^, because diagnostic data from preimaginal stages is available only for a few species^16–20^. Molecular data relevant for species identification are limited to eight species, including five species studied in the present paper^5,12,21^. However, available sequences refer to various genes (e.g. cytB, COI) and their limited variation may be insufficient for reliable species identification as has been demonstrated for the flesh fly genus *Sarcophaga* Meigen^22^.

**Figure 1:**
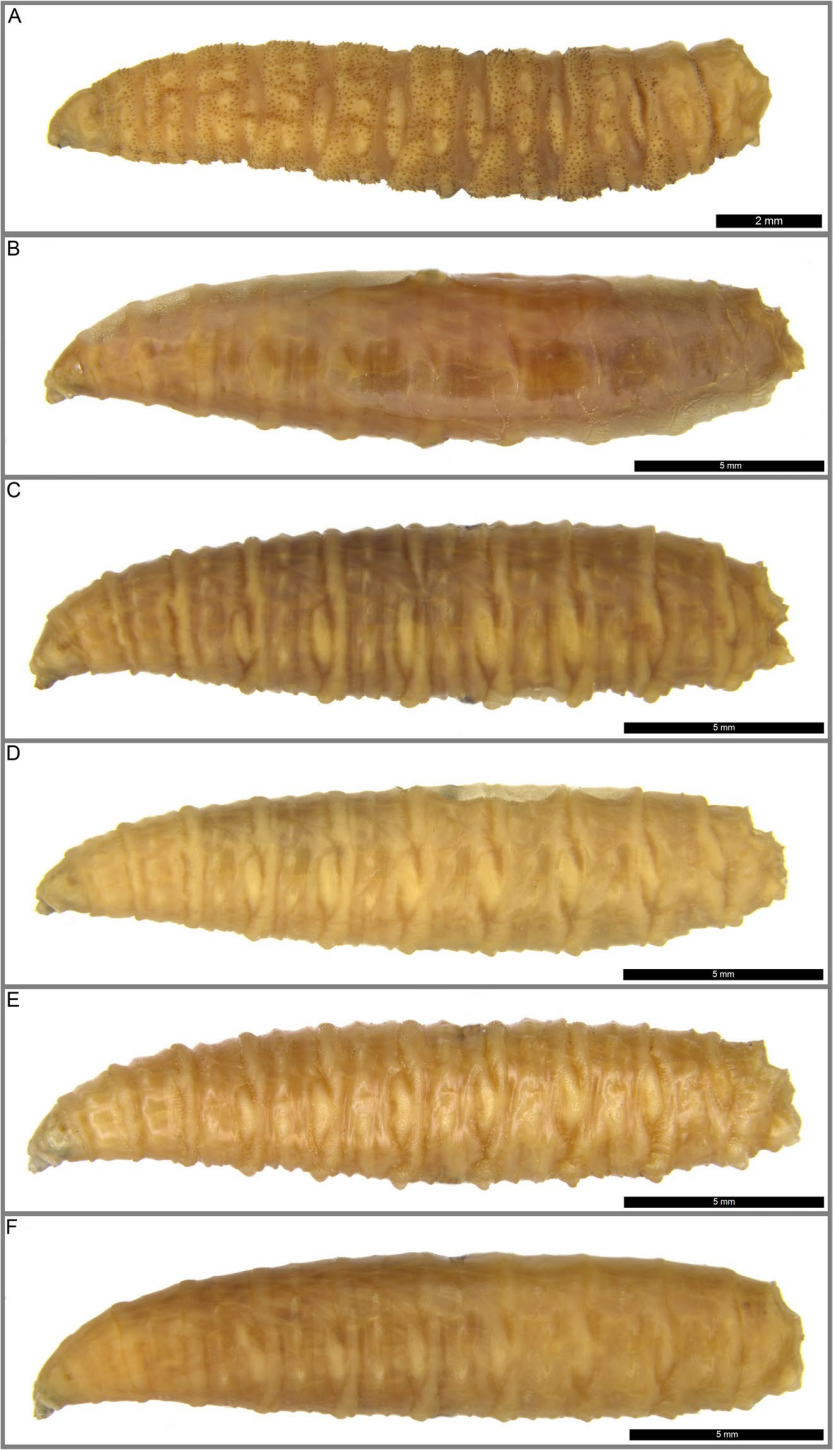
Habitus of *Wohlfahrtia* larvae studied: (**A**) - *W. magnifica*, (**B**) *- W. bella*, (**C**) - *W. indigens*, (**D**) - *W. nuba*, (**E**) - *W. trina*, (**F**) - *W. villeneuvi*.

The method of comparing muscle attachment site (MAS) patterns has proved to be powerful in late instar larvae of forensically important Calliphoridae^23–25^ and Sarcophagidae^26^. The aim of this study was to further extend the method for the six most common species of the western Palaearctic *Wohlfahrtia* (Fig. 1). The species were chosen because they are known to be obligate or facultative myiasis agents and necrophages with confirmed or potential forensic importance. Furthermore, different feeding habits of facultative parasites/necrophages and obligate parasitic larvae (*W. magnifica*) could be reflected in diverging MAS patterns.

## Results

**Figure 2:**
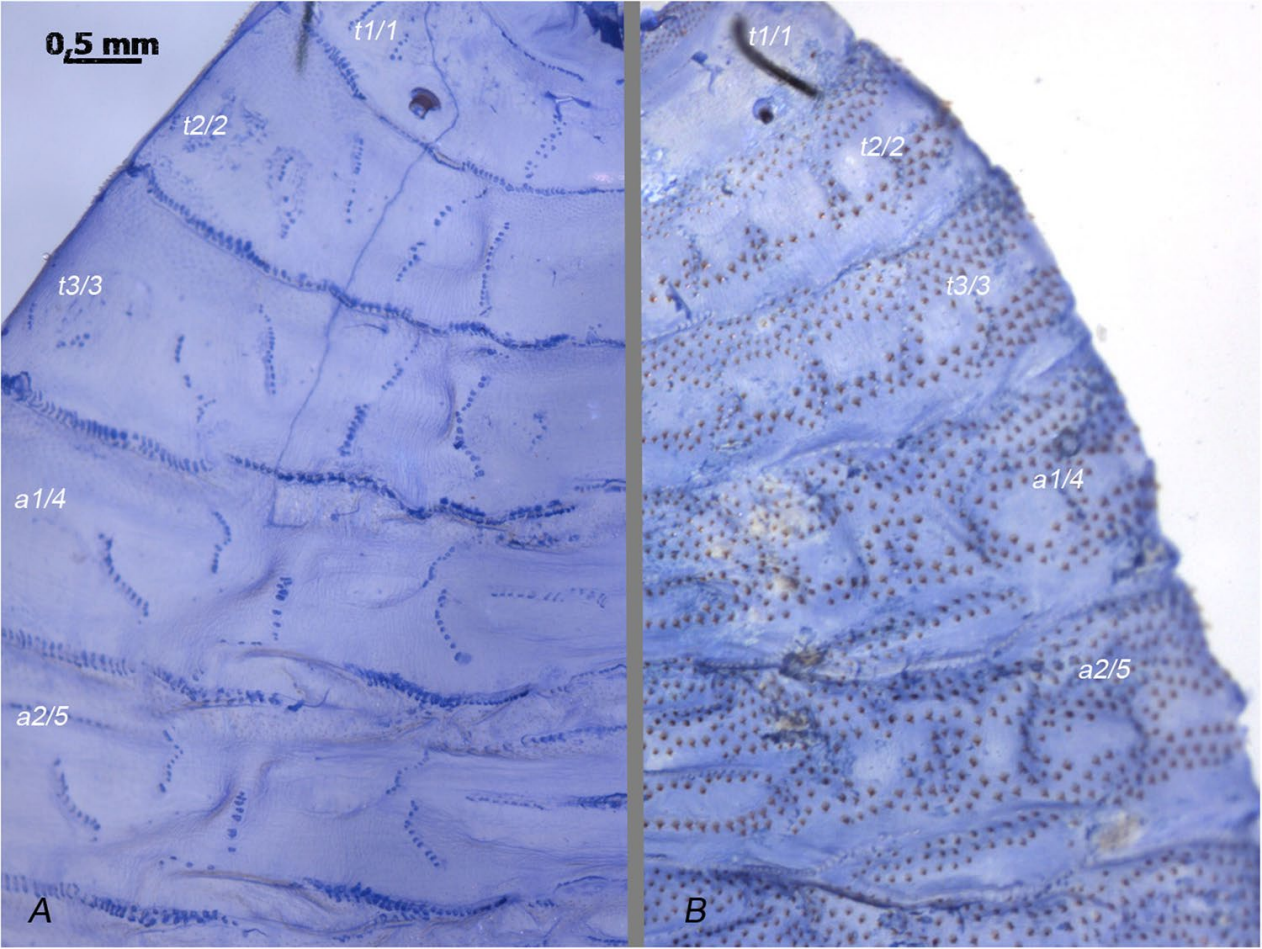
Segments 2–5 of *Wohlfahrtia* third instar larvae, cuticula only, dyed with Coomassie-brillant blue solution 1%: (**A**) - *W. trina*, MAS pattern clearly visible, (**B**) – *W. magnifica*, MAS pattern obscured by sclerotized spines, t = thoracic segment, a = abdominal segment and numbers for plain designation.

The number of MAS for body segments 2–4, (i.e., thoracic segments 2–3 and abdominal segment 1) (Fig. 2), ranged from 171 to 234 (Table 1). The lowest total number of MAS was found in an individual of *W. indigens* and the highest number was found in *W. trina*. The smallest individuals investigated belonged to *W. indigens*, and the largest to *W. bella* (Table 1). Individuals of *Wohlfahrtia magnifica* (Schiner, 1862) (screwworm fly) (n = 13), the only obligate parasite included in this study, could not be analyzed as sclerotized spines on the cuticle obscured the MAS pattern (Fig. 2B).

**Table 1:** Average size of *Wohlfahrtia* larvae (±STD) compared to the ranges in numbers of MAS in 3 hemisegments.

	*W. bella*	*W. indigens*	*W. nuba*	*W. trina*	*W. villeneuvi*
Mean length [mm]	19.11 (±1.52)	15.16 (±0.96)	16.77 (±1,06)	18.57 (±0.95)	17.11 (±1.3)
#MAS in 3 hemisegments	191–223	171–206	175–206	190–234	174–219

The average number of MAS per hemisegment over all larvae was 55 (±6) for segment 2, 66 (±6) for segment 3 and 77 (±6) for segment 4. The numbers of MAS in pattern rows are consistent in most cases (Table 2). Rows with deviations (*framed*) can therefore be of important assistance for species determination.

**Table 2:** Average numbers of MAS per row structure for five *Wohlfahrtia* species and an average for the genus (±STD). Italic values indicate most obvious deviations from genus pattern numbers.

Row	2.1	2.2	2.3	2.4	2.5		
Genus	2 (±1.1)	16 (±2.1)	15 (±2.7)	12 (±1,9)	11 (±2.1)		
*W. bella*	2 (±0.8)	16 (±1.1)	*17 (±1.3)*	13 (±1.2)	10 (±1.5)		
*W. indigens*	2 (±0.6)	14 (±1.4)	14 (±2.4)	12 (±2.2)	11 (±0.7)		
*W. nuba*	*0 (±0.0)*	14 (±0.5)	15 (±2.4)	12 (±1.9)	10 (±0.5)		
*W. trina*	3 (±1.0)	18 (±2.1)	15 (±3.2)	12 (±1.6)	*13 (±2.1)*		
*W. villeneuvi*	2 (±1.4)	15 (±2.0)	14 (±2.5)	13 (±2.3)	11 (±2.5)		
**Row**	**3.1**	**3.2**	**3.3**.	**3.4**	**3.5**		
Genus	6 (±0.9)	19 (±2.2)	17 (±2.5)	13 (±1.7)	12 (±1.3)		
*W. bella*	7 (±0.7)	19 (±1.5)	19 (±2.2)	*15 (±1.7)*	12 (±0.8)		
*W. indigens*	6 (±0.8)	18 (±2.3)	17 (±2.6)	12 (±1.4)	12 (±1.3)		
*W. nuba*	5 (±0.8)	*17 (±1.7)*	15 (±1.4)	13 (±1.5)	12 (±0.9)		
*W. trina*	5 (±0.6)	19 (±2.2)	18 (±2.8)	13 (±1.7)	13 (±1.4)		
*W. villeneuvi*	6 (±0.8)	19 (±2.5)	16 (±2.0)	12 (±1.3)	12 (±1.2)		
**Row**	**4.1a_center**	**4.1a_side**	**4.1b**	**4.2**	**4.3**	**4.4**	**4.5**
Genus	7 (±2.5)	9 (±1.5)	6 (±1.7)	25 (±2.4)	14 (±2.2)	11 (±1.8)	13 (±1.5)
*W. bella*	7 (±1.9)	9 (±1.2)	7 (±1.3)	26 (±2.3)	*16 (±1.1)*	12 (±1.1)	13 (±1.8)
*W. indigens*	7 (±2.3)	7 (±1.0)	6 (±1.4)	24 (±1.8)	13 (±1.1)	10 (±0.7)	12 (±1.2)
*W. nuba*	8 (±2.7)	7 (±1.2)	6 (±0.9)	*22 (±2.5)*	13 (±1.1)	11 (±0.8)	12 (±1.0)
*W. trina*	8 (±3.1)	9 (±1.9)	6 (±1.0)	26 (±1.9)	15 (±2.7)	11 (±1.4)	13 (±1.2)
*W. villeneuvi*	7 (±3.0)	9 (±1.2)	7 (±3.0)	25 (±2.9)	14 (±1.0)	11 (±1.6)	13 (±1.7)

Deviations in pattern rows usually showed no more than two MAS above or below the average count for the genus. Deviations above average were predominantly found in *W. bella*, which comprised the largest larvae investigated. *Wohlfahrtia indigens*, comprising the smallest larvae, showed only one and *W. villeneuvi* showed no such deviations (Table 2). Only one deviation was found with three MAS below the average genus count. This observation was made in *W. nuba*, which also presented five locations with deviations of 2 MAS below average count for the genus.

The genus pattern, generated from five species of the genus *Wohlfahrtia* investigated in this study, is the base of comparison for the researcher in order to distinguish larvae of *Wohlfahrtia* from larvae of *Sarcophaga*. The species can then be identified using the species patterns which are described in comparison to the genus pattern in the following.

**Figure 3:**
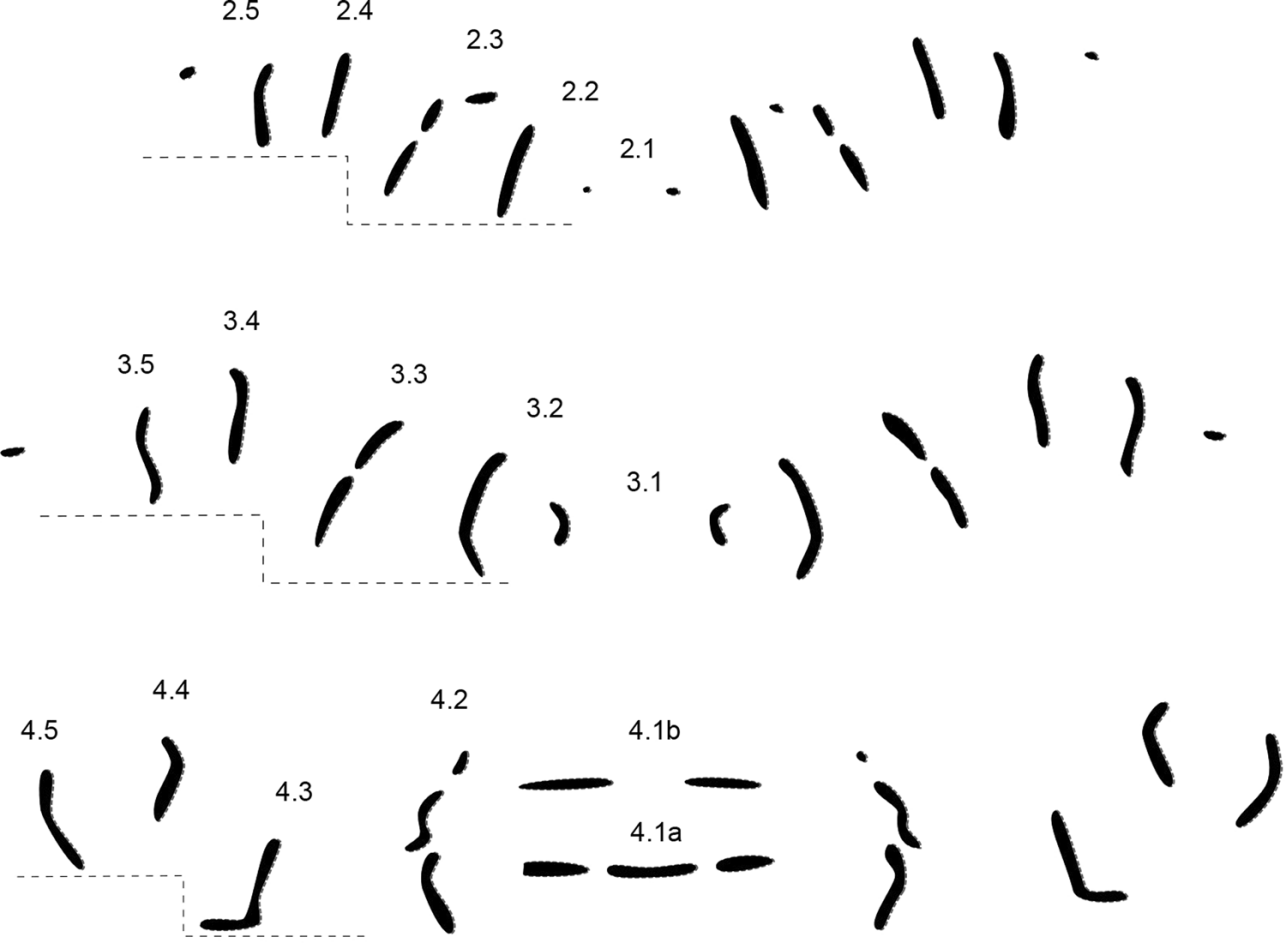
Genus pattern for *Wohlfahrtia* composed of 56 individual MAS patterns of *W. bella, W. indigens, W. nuba, W. trina, W. villeneuvi*. Rows are labeled according to their location on and affiliation with a segment (e.g. 2.1 = central row in segment 2, 4.5 = most peripheral row in segment 4), dashed line = step-like appearance of pattern.

Genus pattern (n = 56) (Fig. 3).

The genus pattern contains MAS in all numbered rows of segments 2–4 (or thoracic segments 2–3 and abdominal segment 1), no void spaces are present. Row 2.1 is very short and dot-like in appearance. Rows 2.2, 2.4 and 2.5 are short vertical rows, whereas row 2.3 is tripartite with a bend in the upper half. Rows 2.4 and 2.5 are shorter compared to rows 2.3, giving the overall pattern a step-like appearance.

The central rows 3.1 in segment 3 are pointing their convex parts at each other like inverted brackets while row 3.2 encloses the two small patterns like big closing brackets. Row 3.3 is almost straight with an angle of about 30 degrees to the midline. 3.4 is z-shaped on the left hemisegment but s-shaped on the right hemisegment, whereas 3.5 is the opposite: s-shaped on the left and z-shaped on the right side. The step-like appearance is maintained as in segment 2.

4.1b comprises two short mirrored horizontal rows in the middle. 4.1a is composed of two short mirrored horizontal rows divided by a small horizontal row in the middle. Rows 4.2 are again tripartite similar to rows 2.3. 4.3 is L-shaped, 4.4 and 4.5 are almost mirrored bent vertical rows but 4.5 is longer than 4.4. The step-like appearance is again maintained in this segment as well.

**Figure 4:**
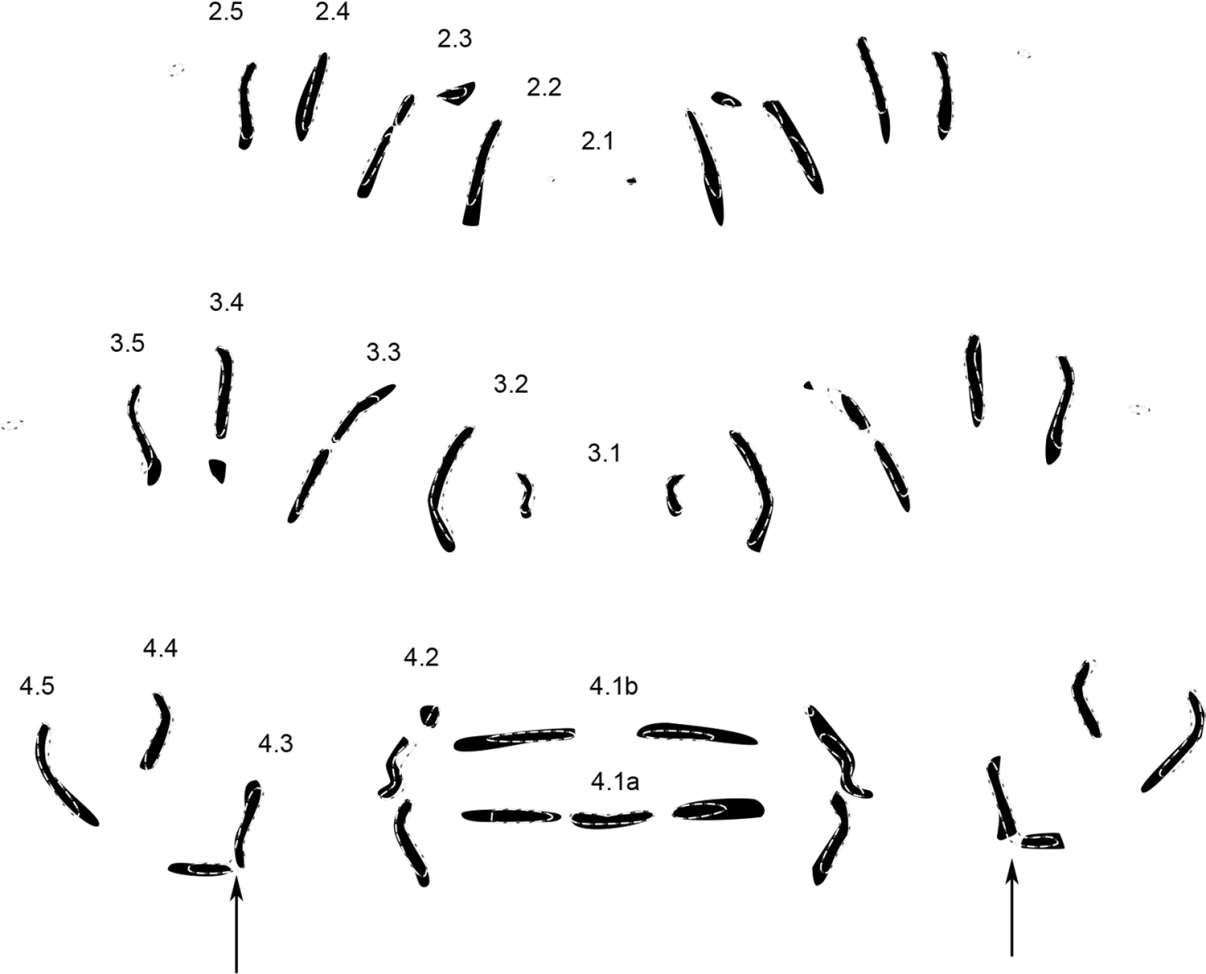
Condensed MAS pattern for *Wohlfarthia bella* (solid structures) superimposed with outlines of the genus pattern (dotted lines). *Numbers* indicate rows of transversal muscle attachment site patterns according to their location on and affiliation with a segment. *Arrows* indicate important differences compared to genus pattern.

*Wohlfahrtia bella* (Macquart, 1839) (n = 11) (Fig. 4).

The most obvious difference of *W. bella* compared to the genus pattern is a separation in rows 4.3 (arrows). The other rows correspond to the genus pattern, although variations can be found in one or the other half of a segment, but not in both.

**Figure 5:**
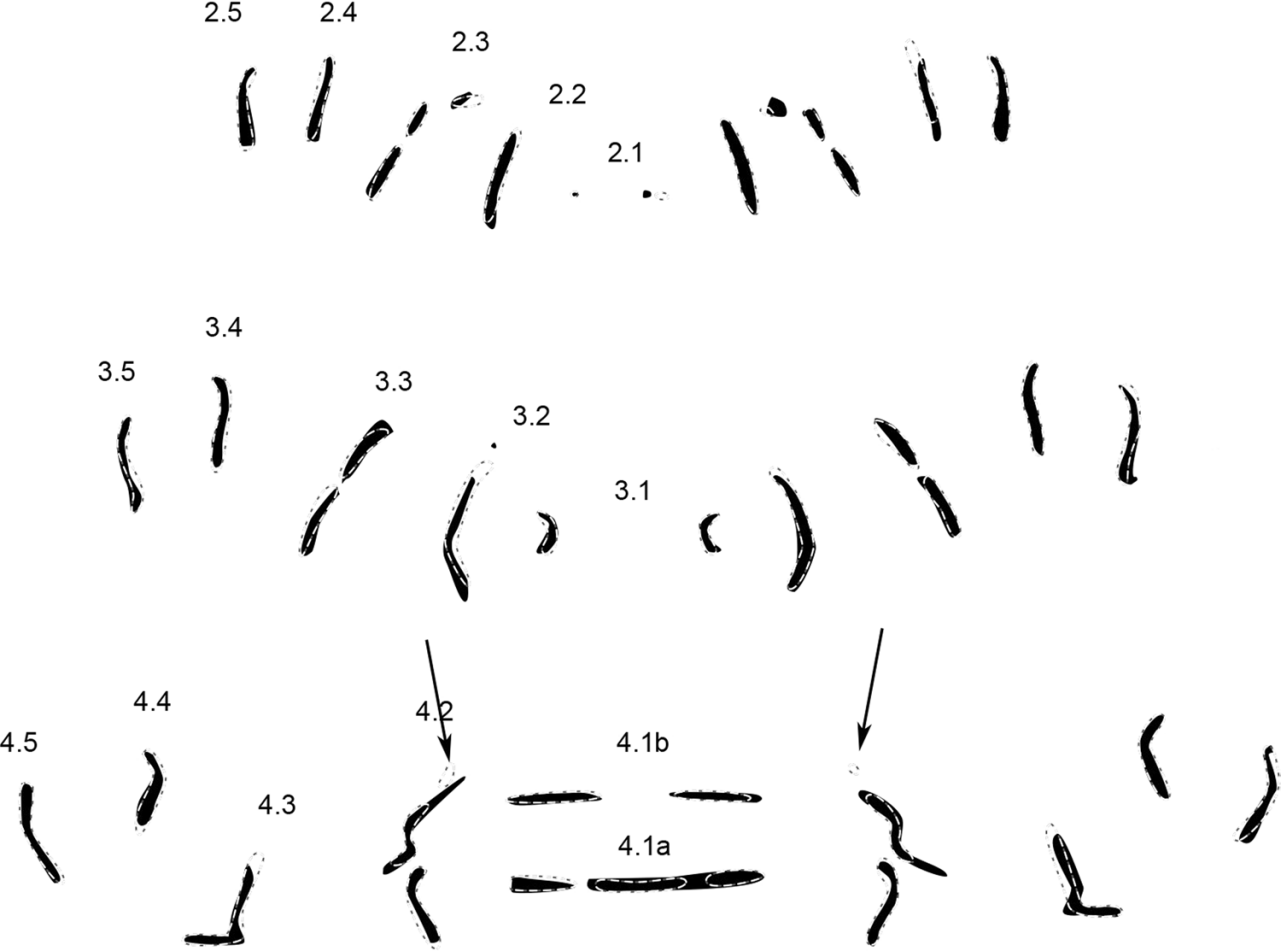
Condensed MAS pattern for *Wohlfarthia indigens* (solid structures) superimposed with outlines of the genus pattern (dotted lines). *Numbers* indicate rows of transversal muscle attachment site patterns according to their location on and affiliation with a segment. *Arrows* indicate important differences compared to genus pattern.

*Wohlfahrtia indigens* Villeneuve, 1928 (n = 10) (Fig. 5).

Central row 4.1a lacks a distinct division between the center and the right side. Row 4.2 has only two, not three parts (arrows), eluding the trisection as seen in the genus pattern. The other rows of all segments line up almost perfectly with the genus pattern.

**Figure 6:**
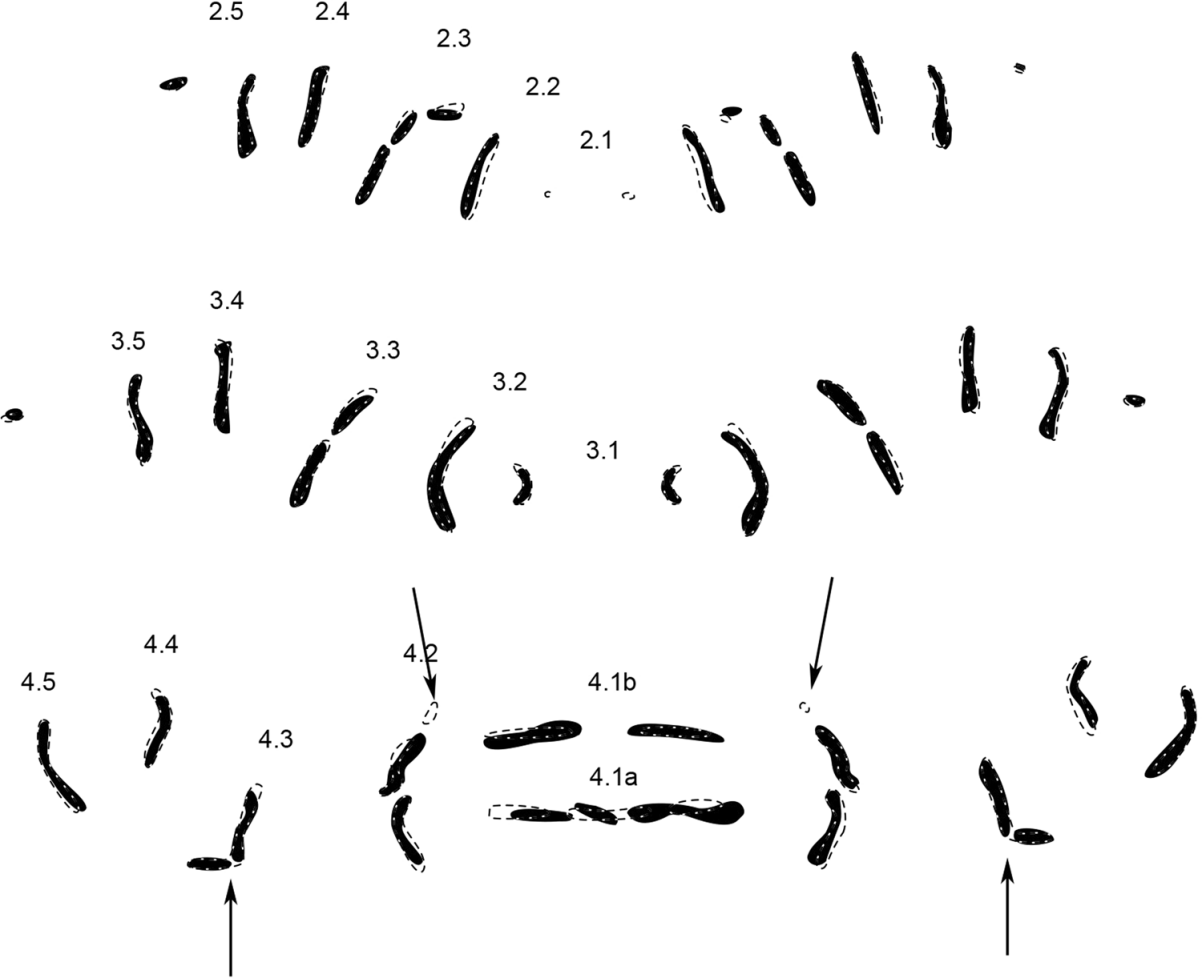
Condensed MAS pattern for *Wohlfarthia nuba* (solid structures) superimposed with outlines of the genus pattern (dotted lines). *Numbers* indicate rows of transversal muscle attachment site patterns according to their location on and affiliation with a segment. *Arrows* indicate important differences compared to genus pattern.

*Wohlfahrtia nuba* (Wiedemann, 1830) (n = 11) (Fig. 6).

*Wohlfahrtia nuba* is almost a combination of *W. bella* (Fig. 3) and *W. indigens* (Fig. 4), as both distinctions of these species are present. The top parts of row 4.2 are missing (arrows), eluding the trisection as seen in the genus pattern, and rows 4.3 have a separation (arrows). Furthermore, row 2.1 is completely missing in *W. nuba*.

**Figure 7:**
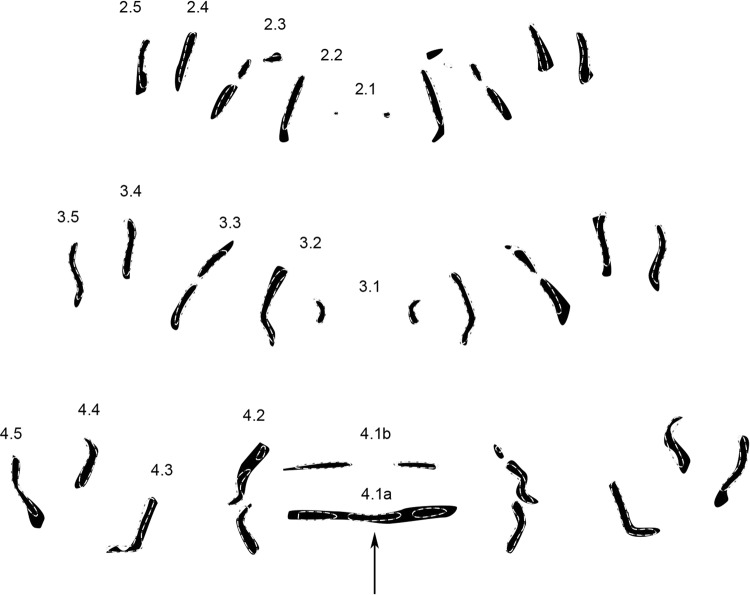
Condensed MAS pattern for *Wohlfarthia trina* (solid structures) superimposed with outlines of the genus pattern (dotted lines). *Numbers* indicate rows of transversal muscle attachment site patterns according to their location on and affiliation with a segment. *Arrows* indicate important differences compared to genus pattern.

*Wohlfahrtia trina* (Wiedemann, 1830) (n = 12) (Fig. 7).

In *W. trina*, similar to *W. villeneuvi* (Fig. 7) and *W. indigens* (Fig. 4), row 4.1a is not tripartite as in the genus pattern. In *W. trina* however, the parts of 4.1a have merged into a single row without any separation. Row 2.2 is longer than in the genus pattern. Other differences are not consistent between the two sides of the segments.

**Figure 8:**
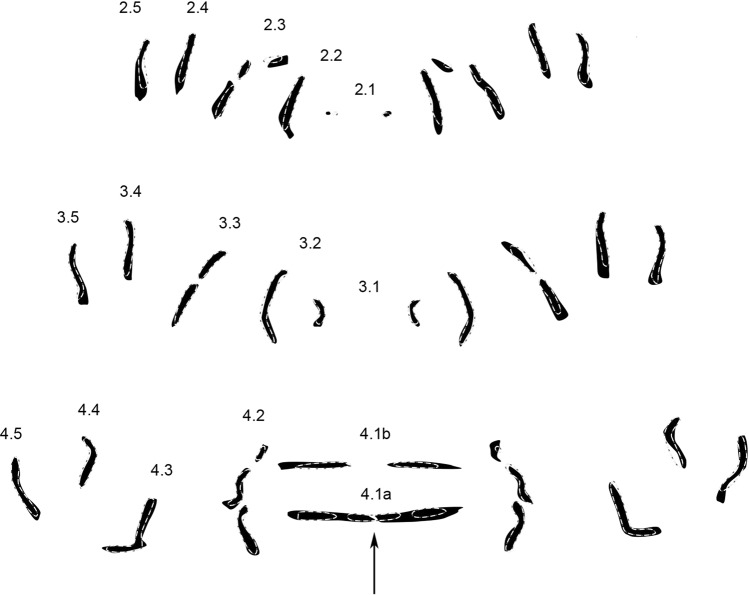
Condensed MAS pattern for *Wohlfarthia villeneuvi* (solid structures) superimposed with outlines of the genus pattern (dotted lines). *Numbers* indicate rows of transversal muscle attachment site patterns according to their location on and affiliation with a segment. *Arrows* indicate important differences compared to genus pattern.

*Wohlfahrtia villeneuvi* Salem, 1938 (n = 12) (Fig. 8).

The most obvious difference in *W. villeneuvi* as compared to the genus pattern can be found in row 4.1a, where the partition is in the middle of two parts instead of a trisection with one central part. Almost all rows of segment 2 are slightly longer than in the genus pattern and row 2.2 is more curved. The rows of segment 3 correspond to those of the genus pattern.

### Comparison of *Wohlfahrtia* and *Sarcophaga*

**Figure 9:**
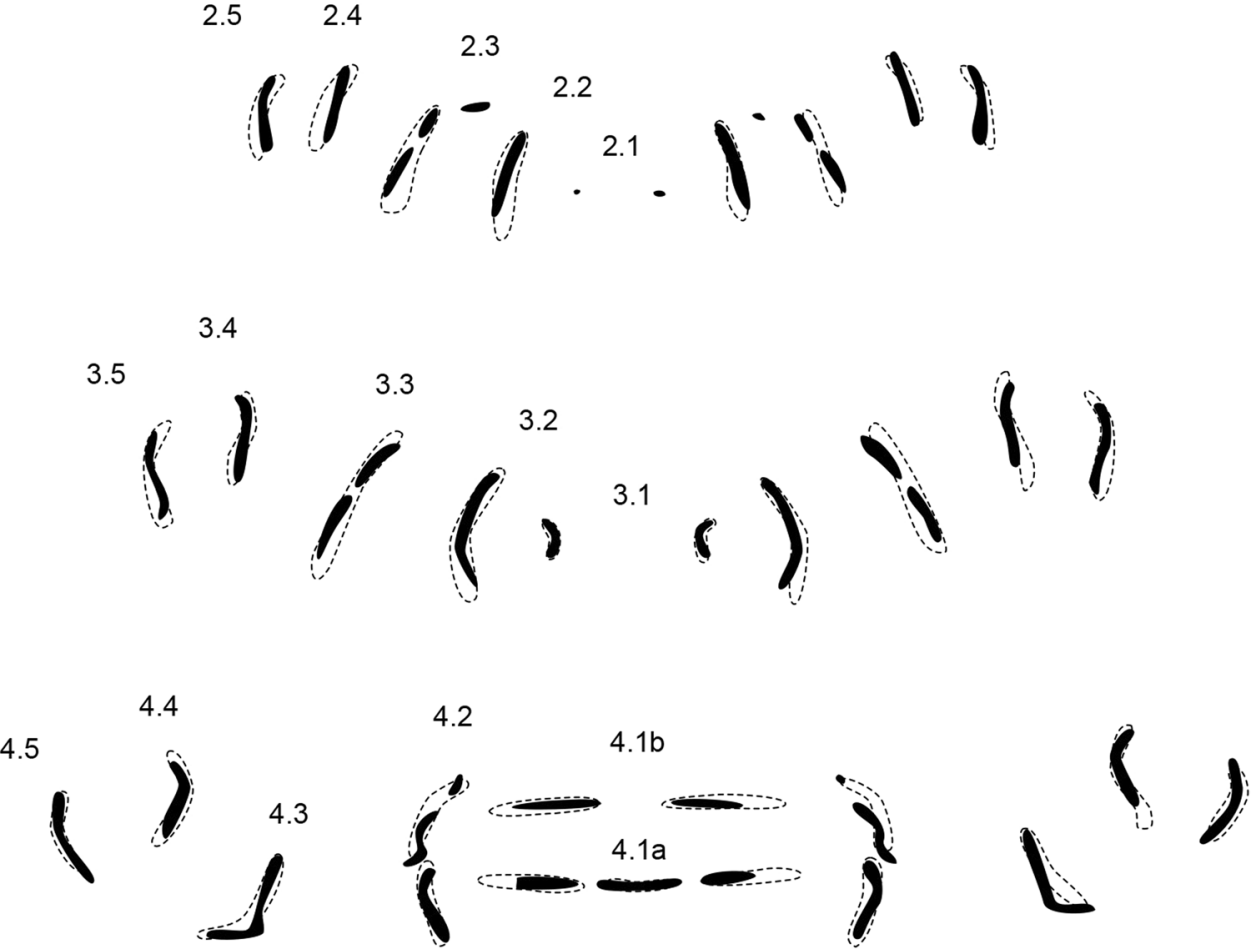
Genus pattern of *Wohlfarthia* (solid structures) superimposed with outlines of the genus pattern of *Sarcophaga* (dotted lines). *Numbers* indicate rows of transversal muscle attachment site patterns according to their location on and affiliation with a segment.

In comparison to the genus pattern of *Sarcophaga* (see Niederegger *et al*. 2016^26^), the rows appear shorter and narrower in the genus pattern of *Wohlfahrtia*. Nevertheless, the number of MAS is significantly higher in all rows in *Wohlfahrtia* (non-parametric Mann-Whitney U test, p < 0.05) (Table 3) with an average total number of MAS for three hemisegments of 206 in *Wohlfahrtia* and 163 in *Sarcophaga*. The trisection of rows 2.3 and 4.2 as well as the bisection of row 3.2 are the most obvious differences between the genera (Fig. 9).

**Table 3:** Comparison of average MAS numbers in rows of *Wohlfarthia* (*W*.) and *Sarcophaga* (*S*.). All differences are significant (non-parametric Mann-Whitney U test).

Row	2.1	2.2	2.3	2.4	2.5		
Genus *W*.	2 (±1.1)	16 (±2.1)	15 (±2.7)	12 (±1,9)	11 (±2.1)		
Genus *S*.	0 (±0.5)	12 (±2.6)	13 (±2.9)	11 (±2.7)	8 (±1.8)		
**Row**	**3.1**	**3.2**	**3.3**.	**3.4**	**3.5**		
Genus *W*.	6 (±0.9)	19 (±2.2)	17 (±2.5)	13 (±1.7)	12 (±1.3)		
Genus *S*.	4 (±1.1)	14 (±2.4)	14 (±2.9)	12 (±2.8)	10 (±2.0)		
**Row**	**4.1a_center**	**4.1a_side**	**4.1b**	**4.2**	**4.3**	**4.4**	**4.5**
Genus *W*.	7 (±2.5)	9 (±1.5)	6 (±1.7)	25 (±2.4)	14 (±2.2)	11 (±1.8)	13 (±1.5)
Genus *S*.	5 (±2.3)	7 (±2.1)	6 (±2.2)	17 (±3.4)	11 (±2.2)	9 (±2.5)	9 (±1.9)

## Discussion

We studied facultative parasites/necrophages and one obligate parasite (*W. magnifica*) a serious traumatic myiasis agent of humans and animals^3^.

The parasite, *W. magnifica*, seems to need additional passive mechanisms to be able to support an obligate parasitic lifestyle, expressed by the bands of sclerotized spines found on the interband surface of segments (Fig. 1A). These spines unfortunately obscure the MAS patterns on the inside of the cuticula, which prevented the analysis (Fig. 2B). In a future endeavor, a method needs to be found to elude such obstacles by clearing pigments or using a different dyeing method. The passive mechanism provided by the spines could even have caused a reduction of MAS.

The remaining five species of the genus *Wohlfahrtia* could be examined and yielded a homogenous genus pattern as well as species specific pattern variations. As in previous studies of MAS patterns, analyses were restricted to the thoracic segments of the third instar larvae. Size differences were reflected somewhat in the numbers of MAS assembling the pattern rows. The individual with the lowest number belonged to the smallest species (*W. indigens*) but the larvae with the highest number did not belong to the largest species (Table 1).

Deviations of MAS numbers from the average genus count for each row were never larger than 2, except for *W. nuba* with one deviation of 3 MAS in Row 4.2. *Wohlfahrtia bella*, the species with the largest larvae had four deviations above average genus count. *Wohlfahrtia nuba*, a facultative parasite had six deviations of 2 or more MAS below average genus count. *Wohlfahrtia villeneuvi*, the one solely necrophagous species analyzed in this study, was not different from her facultative parasitic comrades. With an intermediate size the species also had an intermediate number of MAS and no apparent deviation in MAS numbers from the average (Tables 1 and 2). The only difference to the genus pattern was found in row 4.1a (Fig. 5). Necrophagous and facultative parasitic habits therefore do not seem to have a significant effect on the MAS pattern in *Wohlfahrtia* species.

The genera *Sarcophaga* and *Wohlfahrtia* belong to the family Sarcophagidae. Differentiation is easy in the first instar larvae of Paramacronychiinae and Sarcophaginae as in the second taxon the labrum is vestigial^19^. However, their third instar larvae have very similar external morphology^27^, so the differences in the MAS patterns discovered during the present research are all the more striking. The study presented here is therefore additional evidence for the suitability of MAS analysis for species determination in a variety of cyclorrhaphous Diptera.

For further improvement of the MAS method for species determination in dipteran larvae, the first steps into computerization were conducted in a previous study^28^. This approach will reduce researcher bias and facilitate the method. For a successful advancement, however, the analysis of many more larvae from a broader range of species and genera will be necessary.

## Materials and Methods

### Insects

Adult females of *W. bella, W. indigens, W. nuba, W. trina* and *W. villeneuvi* were attracted to decomposing chicken liver and collected by sweep net. Larvae of obligate parasite *W. magnifica* were collected directly from the host wounds. Detailed data regarding collecting locations in Greece, Iran and Israel are in Table 4. The first instar larvae were obtained by gently squeezing the abdomen of sedated females. Larvae from different females were reared separately in plastic containers (150 ml) with small portions of chicken liver (20–30 gr) as feeding medium. When the larvae reached the third instar they were killed by dousing with boiling water and stored in 70% ethanol. Unambiguous species identification was possible by breeding some larvae of each species to the adult form. Obtained male specimens were identified using the most recent key on the genus^15^. All maternal females and laboratory bred adult specimens were labeled and are available as voucher specimens in the insect collection of the Department of Ecology and Biogeography, Faculty of Biological and Veterinary Sciences, Nicolaus Copernicus University in Toruń, Poland.

**Table 4:** Collecting locations for larvae.

Species	Location	No. of larvae
*Wohlfahrtia bella*	Iran, North Khorasan, Marghzar, 1145 m.a.s.l., 37°03′52″N 56°16′14″E, leg. KEiB Iran Expedition I	8
*Wohlfahrtia bella*	Iran, North Khorasan, Ru’in, 1782 m.a.s.l., 37°11′47″N 57°29′02″E, leg. KEiB Iran Expedition I	3
*Wohlfahrtia indigens*	Iran, Kerman, Anduhjerd, 757 m.a.s.l., 30°14′15″N 57°47′14″E, leg. KEiB Iran Expedition II	3
*Wohlfahrtia indigens*	Iran, Kerman, Shahdad, 420 m.a.s.l., 30°27′39″N 57°43′19″E, leg. KEiB Iran Expedition II	7
*Wohlfahrtia magnifica*	Greece, Thrace, Kardamos, 500 m.a.s.l., 25° 37′ 30″E, 41° 16′ 43″N, leg. S. Sotiraki	7
*Wohlfahrtia magnifica*	Iran, Fars, Dasht Arzan, 1990 m.a.s.l. 51°58′33″N 29°39′21″E, leg. K. Akbarzadeh	6
*Wohlfahrtia nuba*	Iran, North Khorasan, Sarcheshmeh, 932 m.a.s.l., 37°38′00″N 57°24′25″E, leg. KEiB Iran Expedition I	3
*Wohlfahrtia nuba*	Iran, North Khorasan, Darband, 1129 m.a.s.l., 37°14′10″N 56°49′50″E, leg. KEiB Iran Expedition III	11
*Wohlfahrtia trina*	Israel, Ein Avdat NP, Nahal Zin, 328 m.a.s.l., 30°50‘26″N 34°48′35″E, leg. K. Szpila	4
*Wohlfahrtia trina*	Iran, Khorasan-e-Razavi, Mir Haj, 1042 m.a.s.l., 36°39′41″N 56°38′50″E, leg. KEiB Iran Expedition I	4
*Wohlfahrtia trina*	Iran, Khorasan-e-Razavi, Mir Haj, 1042 m.a.s.l., 36°39′41″N 56°38′50″E, leg. KEiB Iran Expedition I	4
*Wohlfahrtia villeneuvi*	Iran, Kerman, Tachrud, 1711 m.a.s.l., 29°23′31″N 57°52′36″E, leg. KEiB Iran Expedition II	7
*Wohlfahrtia villeneuvi*	Israel, Ein Avdat NP, Ein Akev Spring, 404 m.a.s.l., 30°48 16″N 34 48′48″E, leg. K. Szpila	5

### Preparation

The larvae were measured to the nearest 0.1 mm using a dissecting microscope (Zeiss Stemi 2000C) with digital camera (Zeiss AxioCam ICc1) and measuring software (AxioVision). Larvae were opened along the dorsal midline and all muscle layers removed. The central rows of the MAS patterns correspond to the ventral center at the inside of the cuticula of each segment. All subsequent preparation and evaluation steps leading to the condensed patterns were performed as given in our previous publications^23,26,29^.

### Data evaluation

Higher dipteran larvae are composed of a pseudocephalon and additional 11 segments. The first three segments are referred to as thoracic segments (t1–3), the following 7 are abdominal segments (a1–7) and the last is the anal division. For the study of MAS a plain designation of segments by numbers was chosen for convenience and clarity (Fig. 2). All rows in segments 2–4 were labeled according to our previous publication on *Sarcophaga* species (Niederegger *et al*. 2016^26^). The patterns were evaluated using Inkscape v. 0.91 (available under GPLv2+ from https://inkscape.org) and Adobe Photoshop® CS5 (Adobe Systems, Inc., San Jose, CA, USA); means and standard deviations were calculated using Microsoft® Office Excel® 2010.

### Statistical comparison

The *Wohlfahrtia* genus pattern was compared to the *Sarcophaga* genus pattern as both genera belong to the family Sarcophagidae. More than half of the rows showed non-normal distribution in the numbers of MAS per row, the statistical analysis was therefore performed using a non-parametric Mann–Whitney U test for all rows.

## Data Availability

The data that support the findings of this study are available from the corresponding author, SN, upon reasonable request.
